# Endothelial Lipase Plasma Levels are Increased in Patients With Significant Carotid Artery Stenosis and History of Neurological Impairment

**DOI:** 10.4021/jocmr734w

**Published:** 2012-01-17

**Authors:** Monika Riederer, Matias Trbušić, Vesna Degoricija, Saša Frank

**Affiliations:** a Institute of Molecular Biology and Biochemistry, Center of Molecular Medicine, Medical University of Graz, Graz, Austria; b University of Zagreb School of Medicine, Department of Medicine, Sisters of Mercy University Hospital, Zagreb, Croatia

## Abstract

**Background:**

Endothelial lipase (EL) is a phospholipase expressed predominantly by vascular endothelial cells. The goal of the present study was to examine whether EL plasma levels in patients with carotid artery stenosis differ between those with previous history of neurological impairment and those without neurological symptoms.

**Methods:**

EL plasma levels were measured by a competitive ELISA assay.

**Results:**

EL plasma levels were significantly higher in the symptomatic, compared with the asymptomatic group (mean 489.61 ± 145 ng/ml (n = 31) vs. 388.39 ± 133 ng/ml (n = 24), t-test, P = 0.011).

**Conclusion:**

We concluded that increased EL plasma levels reflect the patients' overall susceptibility for cerebrovascular events.

**Keywords:**

Atherosclerosis; Carotid artery stenosis; Endothelial lipase; Neurological impairment; Carotid endarterectomy

## Introduction

Endothelial lipase (EL) is a member of the triglyceride lipase gene family, expressed by vascular endothelial cells and to a lesser extend by smooth muscle cells and macrophages [1]. EL lowers high-density lipoprotein (HDL) plasma levels, an independent risk factor for atherosclerosis [2]. EL plasma concentrations are increased in metabolic syndrome and associated with coronary atherosclerosis [3]. Most recently, using immunohistochemistry, we observed higher EL protein expression in symptomatic and unstable carotid plaques, compared with those from patients without neurological symptoms and with stable plaque phenotype [4]. The goal of the present study was to examine whether EL plasma levels in patients with carotid artery stenosis differ between those with previous history of neurological impairment and those without neurological symptoms.

## Methods

Overall 66 patients with significant (70 - 99%) carotid artery stenosis, recruited and described as detailed elsewhere [4], were included in the study. Written informed consent from each patient was obtained prior to the enrollment in the study, which was performed according to Good Clinical Practice and Helsinki Declaration principles. The study was approved by the local Ethics Committee, in accordance with institutional guidelines of the Sisters of Mercy University Hospital, Zagreb. Patients were grouped regarding history of previous neurological impairment into asymptomatic and symptomatic. The symptomatic group (ipsilateral stroke, transient ischemic attack or monocular blindness) comprised patients with symptoms unrelated to carotid artery stenosis (previous symptoms) and patients with symptoms referable to the respective carotid artery (recent symptoms).

EL plasma protein levels were analyzed in 55 out of 66 samples by a competitive ELISA assay [5]. In brief, 5 ng of an N-terminal peptide of human EL (Novus Biologicals, Littleton, CO) were immobilized on a 96-well EIA microtiter plate (Costar). After blocking nonspecific binding sites with 5% bovine serum albumine (BSA), a mixture comprising 50 µl of 1:6 diluted plasma sample and 50 µl of an anti-EL antibody (Novus Biologicals) was applied to the wells and incubated at room temperature for 3 h. After extensive washing, the plate was incubated with horseradish peroxidase (HRP)-labeled goat-anti-rabbit secondary antibody. The amount of antibody bound to the immobilized N-terminal peptide of human EL was determined by the HRP-catalyzed dye development using a colorless substrate (Pierce), on an ELISA-reader. The standard curve was prepared by serial dilution of EL N-terminal peptide. Cross-reactivity with other serum lipases, tested with purified preparations of lipoprotein lipase or hepatic lipase (kindly provided by Dr. G. Olivecrona, University of Umea, Sweden), was negligible. Plasma levels of high-sensitivity C-reactive protein hsCRP, IL-6 and HDL were measured as described [4].

Unpaired Student´s t-test and Mann-Whithney-U test were performed to assess differences among EL, hsCRP, IL-6 and HDL plasma concentrations between symptomatic and asymptomatic groups. The association between quantitative data was evaluated using Spearman-rank correlation analysis. Data were analyzed using PASW 17.02 (IBM, Chicago, IL, USA) software and all p values below 0.05 were considered significant.

## Results

**Figure 1:**
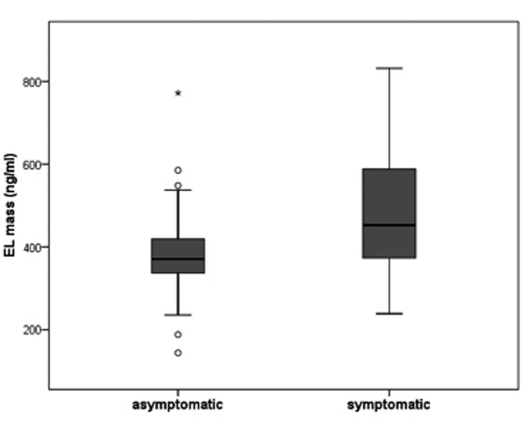
EL protein levels were determined in plasma of 24 asymptomatic and 31 symptomatic patients by competitive ELISA. The boundaries of the box indicate the lower and upper quartiles, the horizontal line the median (370.6 vs. 452.6 ng/ml) and the error bars the 95% confidence interval. Outliers are indicated by ‘°’ and significance is denoted by ‘*’ (P = 0.011).

EL plasma levels were significantly higher in the symptomatic group compared with the asymptomatic group (mean 489.61 ±145 ng/ml (n = 31) vs. 388.39 ± 133 ng/ml (n = 24), t-test, P = 0.011) (Fig. 1). Patients with recent (n = 20) and previous (n = 11) symptoms had similar EL plasma levels. EL plasma levels were not statistically significantly correlated with the markers of inflammation (hsCRP, IL-6) or HDL plasma levels (Spearman correlation; r = 0.15 (P = 0.28) for hsCRP; r = 0.02 (P = 0.88) for IL-6; r = 0.05 (P = 0.72) for HDL); whereas hsCRP and IL-6 levels correlated significantly with each other (r = 0.61; P < 0.01). The hsCRP, IL-6 and HDL plasma levels were similar in symptomatic and asymptomatic patients.

## Discussion

In contrast to plasma levels of inflammatory markers and HDL, the EL plasma levels were significantly higher in symptomatic patients. The lack of association between EL plasma levels and that of inflammatory markers might reflect the impact of gender and age, factors known to modulate plasma levels of inflammatory molecules [6,7]. The lack of significant correlation between plasma EL and HDL levels might be due to medication [8-12] and is in line with a previous report [13]. We concluded that increased EL plasma levels, independent of markers of inflammation or HDL plasma levels, reflect the patients’ overall susceptibility for cerebrovascular events. Whether EL is only a marker or an active causative factor for cerebrovascular events remains to be determined.
